# The feces of sea urchins as food improves survival, growth, and resistance of small sea cucumbers *Apostichopus japonicus* in summer

**DOI:** 10.1038/s41598-023-32226-y

**Published:** 2023-04-01

**Authors:** Yushi Yu, Peng Ding, Yihai Qiao, Yansong Liu, Xiajing Wang, Tongdan Zhang, Jun Ding, Yaqing Chang, Chong Zhao

**Affiliations:** Key Laboratory of Mariculture & Stock Enhancement in North China’s Sea, Ministry of Agriculture and Rural Affairs, Dalian Ocean University, Dalian, China

**Keywords:** Agroecology, Animal behaviour

## Abstract

Mass mortality and low growth highly decrease the production efficiency and sustainable aquaculture development of the sea cucumber *Apostichopus japonicus* in summer. Sea urchin feces was proposed to address the summer problems. A laboratory study was conducted for ~ 5 weeks to investigate survival, food consumption, growth and resistance ability of *A. japonicus* cultured with the feces of sea urchins fed kelp (KF feces, group KF), the feces of sea urchins fed prepared feed (FF feces, group FF), and the prepared sea cucumber feed (group S) at high temperature (25 °C). The sea cucumbers of group KF had better survival (100%) than those of the group FF (~ 84%), higher CT_max_ (35.9 °C) than those of the group S (34.5 °C), and the lowest skin ulceration proportion (0%) when  they were exposed to an infectious solution among the three groups. These results suggest that the feces of sea urchins fed kelp is a promising diet for improving the survival and enhancing the resistance in *A. japonicus* aquaculture in summer. Sea cucumbers fed significantly less FF feces after 24 h of ageing than the fresh FF feces, suggesting this kind of feces became unsuitable for *A. japonicus* in a short time (within 48 h). However, the 24 h of ageing at 25 °C for the high fiber feces of sea urchins fed kelp had no significant effects on the fecal consumption of sea cucumbers. In the present study, both fecal diets provide better individual growth to sea cucumbers than the prepared feed. Yet, the feces of sea urchins fed kelp provided the highest weight gain rate (WGR) to sea cucumbers. Therefore, the feces of sea urchins fed kelp is a promising food to reduce the mortality, to address the problems of summer, and to achieve higher efficiency in *A. japonicus* aquaculture in summer.

## Introduction

The sea cucumber *Apostichopus japonicus* is the most commercially important echinoderm in China, Japan, and Russia^1–3^. However, high temperature aggravates a number of other severe problems, including bacterial infectious diseases, decay of food, and mortality of sea cucumbers, causing considerable economic losses, and resulting in a serious decline in resources^4–6^. Besides, poor growth rate further restricts the development of the aquaculture industry of sea cucumbers in summer^7–9^. Therefore, it is essential to reduce the mortality rate and improve the growth of cultured *A. japonicus* in summer.

The feces of sea urchins, which is rich in active bacteria, digestive enzymes, protozoa and other nutritious organic materials, is a common food for *A. japonicus*^10–13^. Ingesting feces that contains these active substances^14,15^, enhances survival and disease resistance for sea cucumbers^15–18^. Further, sea cucumbers fed feces of sea urchins achieve good growth in the integrated multi-trophic aquaculture (IMTA) system, which had no additional food^18–20^. *Apostichopus japonicus* fed feces had better growth than those fed prepared feed at the optimum temperatures and in winter^20^. Therefore, the feces of sea urchins, as a food with high nutritional value activity organic matter^11,21^, has a great potential to improve the growth of *A. japonicus* in summer*.* It is thus imperative to determine whether fecal diet can solve the problems of sea cucumber aquaculture in summer.

Feces of sea urchins fed kelp is a standard fecal category, because kelp is a common food for sea urchins^22^. Further, the feces of sea urchins fed prepared feed is a good source of food for sea cucumbers at the optimum temperature and in winter^20^. Due to the active substances in the feces, these two kinds of feces have different and specific characteristics after excretion, in which the nutritive value of feces changes complexly over time^11^. The nutritive value of feces usually increases, then decreases, and eventually loses its nutritional value^11^. Considering this process would be affected by the different fecal composition^12^ and sped up by high water temperature in summer, it is important to examine the influence of fecal composition on the usability at high temperature.

The present study investigated that whether a diet of sea urchin feces improves the survival, growth, and resistance of *A. japonicus* in summer. We further explored the effect of different fecal diets on feeding behavior of *A. japonicus* at high temperature, to determine the usability of fecal diets and how nutritional composition affects the usability of fecal diets in summer.

## Results

### Survival rate

**Figure 1:**
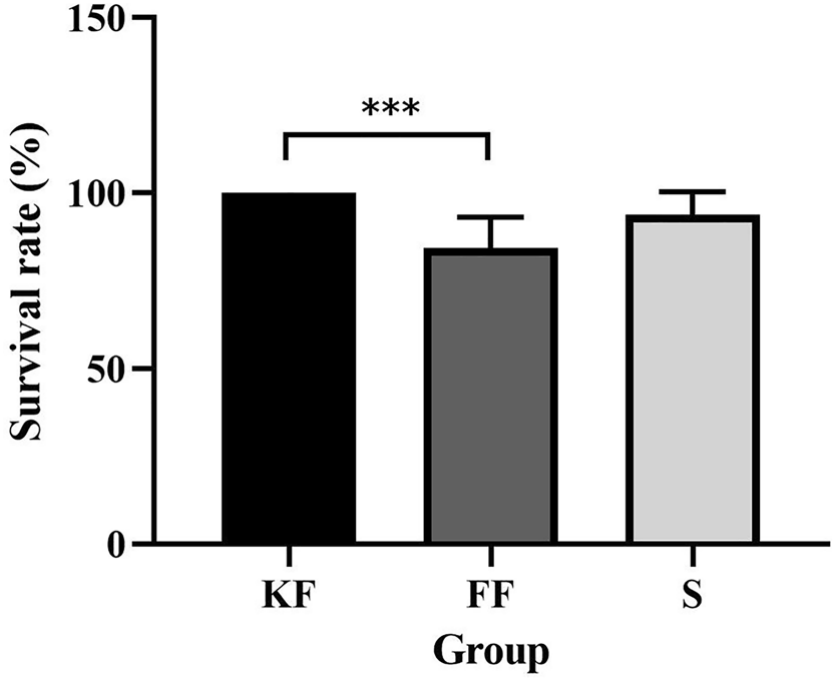
Survival rate of the three group after 5-weeks culture (mean ± SD, N = 8). The asterisk *** means *P* < 0.001.

Diets significantly affected survival rate of sea cucumbers (Kruskal–Wallis = 12.620, *P* = 0.002). The sea cucumbers of group KF (100 ± 0.00%) had significantly higher survival rate than those of group FF (84.38 ± 8.84%, *P* < 0.001). The survival rate of sea cucumbers of group KF was not significantly different from that of group S (91.06 ± 9.11%, *P* = 0.098). There was no significant difference in survival rate between the groups FF and S (*P* = 0.058, Fig. 1).

### Food consumption

**Figure 2:**
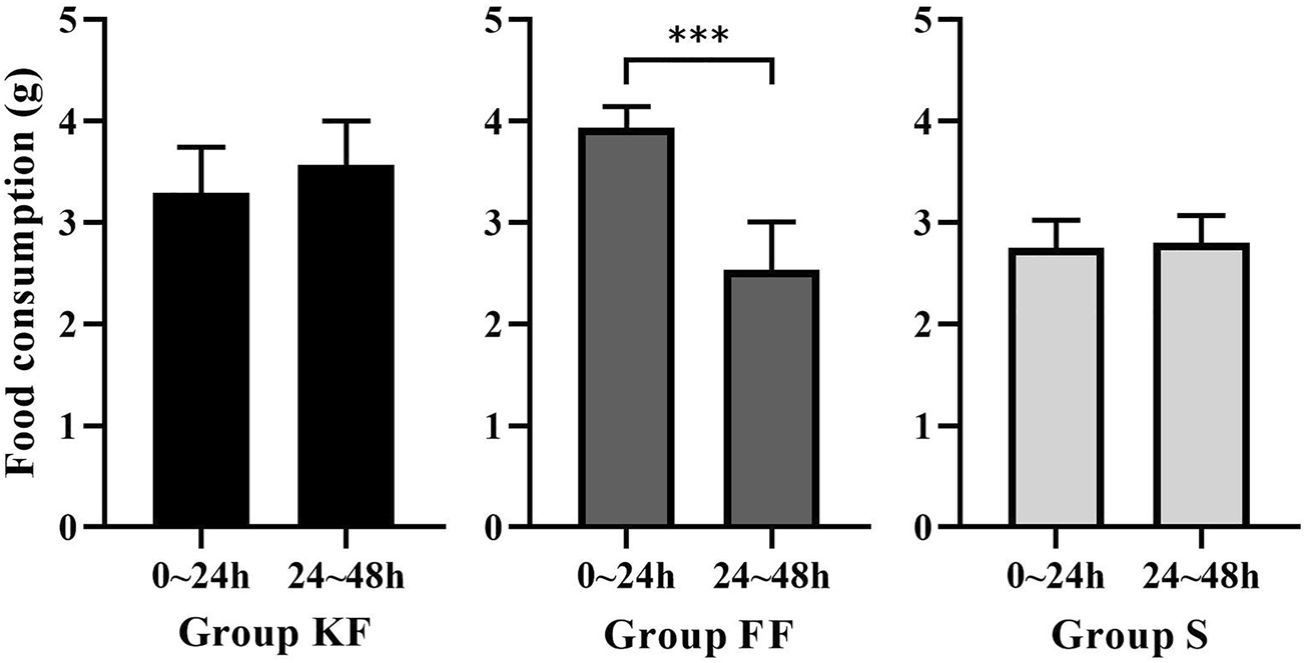
Food consumption of *Apostichopus japonicus* fed fresh food and food aged for 24 h for the three groups (mean ± SD, N = 8). KF, FF, S refer to group KF (consume the feces of sea urchins fed kelp), group FF (consume the feces of sea urchins fed prepared feed) and group S (consume the sea cucumber feed). The asterisk ***means *P* < 0.001.

Food consumption showed no significant difference between sea cucumbers fed fresh KF feces (3.29 ± 0.42 g) and fed KF feces aged for 24 h (3.57 ± 0.41 g) (Mann–Whitney U = 45.500, *P* = 0.161). Sea cucumbers consumed significantly less FF feces aged for 24 h (2.53 ± 0.44 g) than fresh FF feces (3.94 ± 0.20 g) (Mann–Whitney U = 0.000, *P* < 0.001). Food consumption showed no significant difference between the sea cucumbers fed fresh sea cucumber feed (2.75 ± 0.26 g) and aged sea cucumber feed (2.80 ± 0.25 g) (F = 0.033, *P* = 0.707, Fig. 2).

### Growth

**Figure 3:**
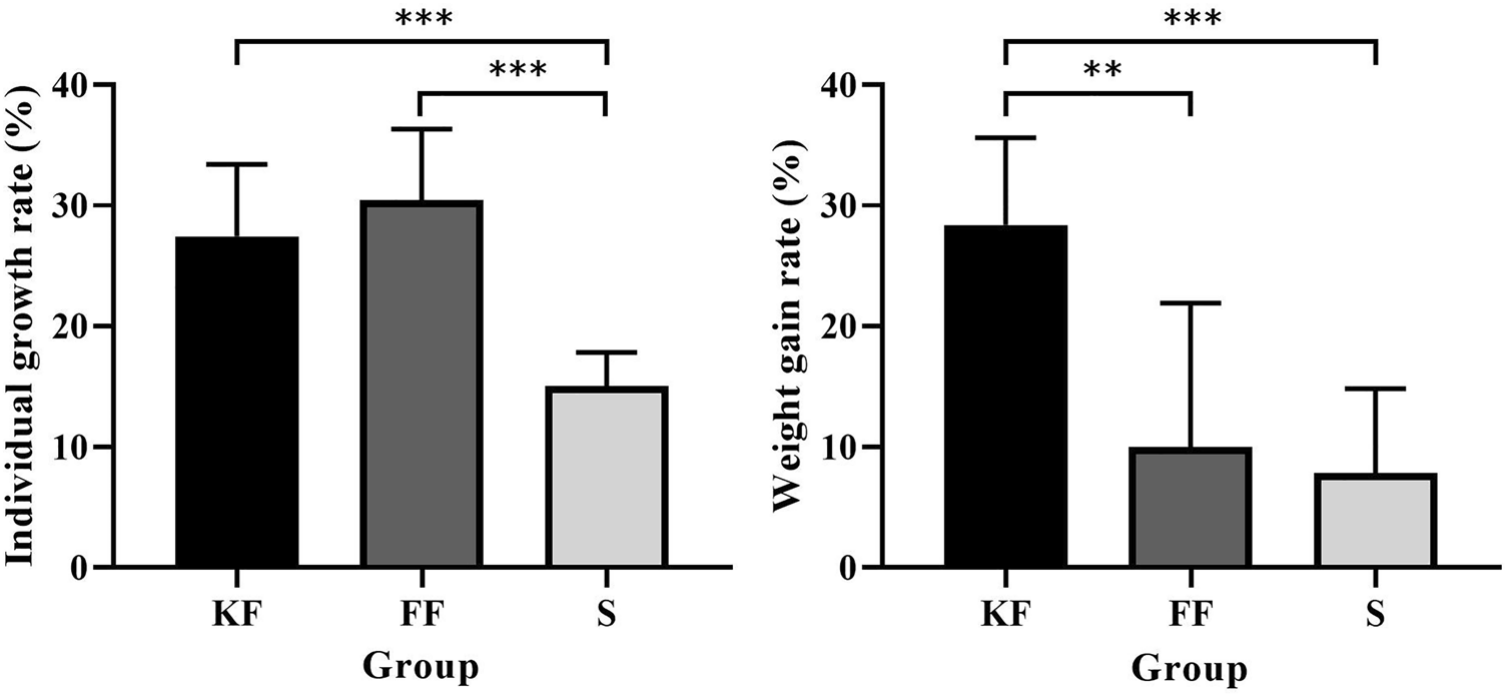
Individual growth rates of *Apostichopus japonicus* in the three groups (mean ± SD, N = 8, left). Weight gain rate (WGR) of *Apostichopus japonicus* in the three groups (mean ± SD, N = 8, right). KF, FF, S refer to group KF, group FF and group S. The asterisks ** and *** mean *P* < 0.01 and *P* < 0.001, respectively.

Growth of individual sea cucumbers in groups KF (27.43 ± 5.60%) and FF (30.46 ± 5.50%) were significantly higher than that in group S (15.09 ± 2.56%, *P* < 0.001 for group KF and group FF, Fig. 3, left).

Weight gain rate (WGR) of individual sea cucumbers of group KF (28.40 ± 6.75%) was significantly higher than that of the other two groups (*P* = 0.009 for group FF, *P* < 0.001 for group S). No significant difference was found between groups FF (10.02 ± 11.12%) and S (7.83 ± 6.55%, *P* = 0.962; Fig. 3, right).

### Skin ulceration proportion

**Figure 4:**
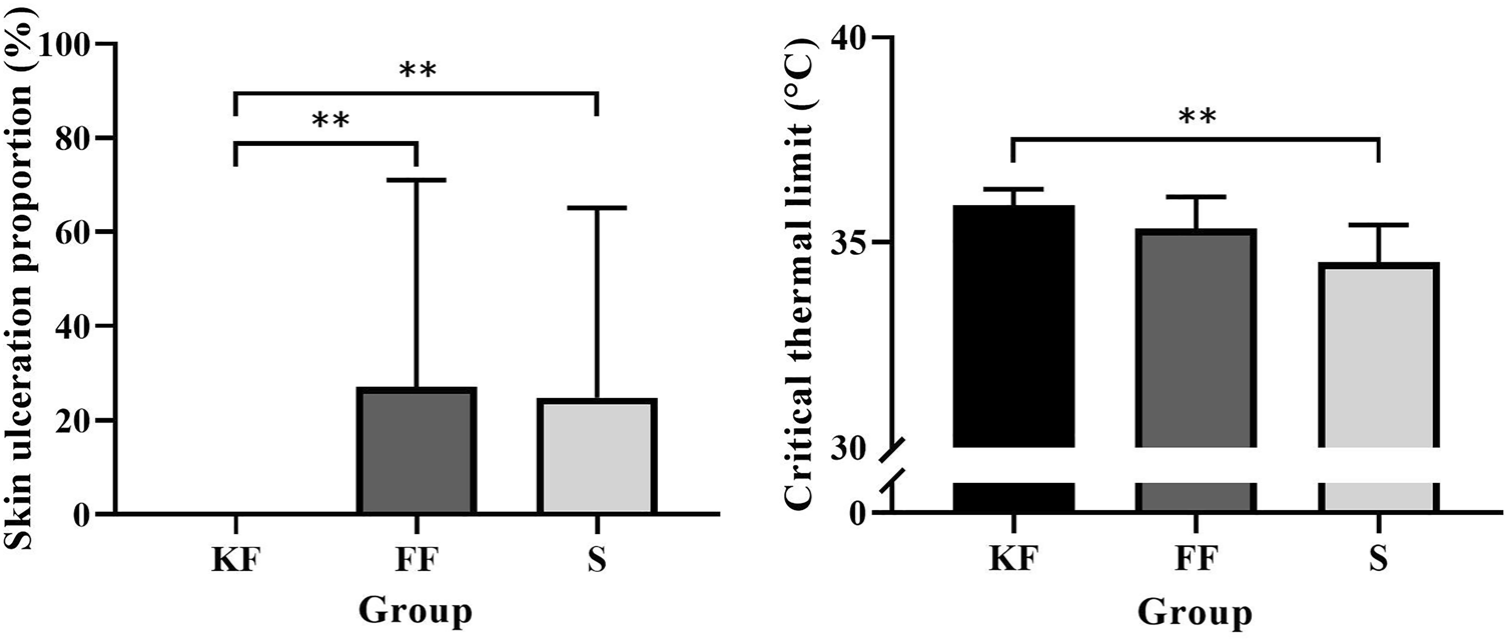
Skin ulceration proportion of *Apostichopus japonicus* covered with ulcers after the exposure to infectious solution in the three groups (mean ± SD, N = 20, left). CT_max_ of *A. japonicus* in the three groups (mean ± SD, N = 12, right). KF, FF, S refer to group KF, group FF and group S. The asterisk ** means *P* < 0.01.

Skin ulceration proportion (per sea cucumber) was significantly lower in group KF (0.00 ± 0.00%) than in groups FF (27.14 ± 42.77%; df = 19, Z = 10.950, *P* = 0.009) and S (24.90 ± 39.24%; df = 19, *Z* = 11.550, *P* = 0.006). There was no significant difference in skin ulceration proportion between groups FF and S (df = 19, Z = 0.600, *P* = 0.886; Fig. 4, left).

### The critical thermal maximum (CT_max_)

CT_max_ of sea cucumbers fed KF feces (35.90 ± 0.38 °C) was significantly higher than in those fed sea cucumber feed (34.52 ± 0.87 °C, F = 10.966, *P* = 0.001). There was no significant difference in CT_max_ between groups KF and FF (35.33 ± 0.74 °C, *P* = 0.109), and between groups FF and S (*P* = 0.081; Fig. 4, right).

### Crude protein, fat and fiber concentration of the three diets

The concentration of crude protein of FF feces (14.31 ± 1.00% dry weight) was significantly higher than the other two diets (*P* < 0.001 for KF feces, *P* < 0.001 for sea cucumber feed). Sea cucumber feed (3.43 ± 0.17% dry weight) and the KF feces (2.42 ± 0.37% dry weight) had no significant difference in concentration of crude protein (*P* = 0.157). The sea cucumber feed has the significantly higher fat concentration (1.40 ± 0.03% dry weight) than the KF feces (*P* = 0.009). KF feces (0.62 ± 0.11% dry weight) and FF feces (0.80 ± 0.02%) dry weight, FF feces and sea cucumber feed had no significant difference in fat concentration (*P* = 0.229, *P* = 0.153). The feces of sea urchins fed kelp (KF) has the highest fiber concentration among the three diets (21.31 ± 0.98% dry weight, *P* < 0.001). The feces of sea urchins fed prepared feed (10.02 ± 0.32% dry weight) had significantly higher fiber concentration than sea cucumber feed (2.15 ± 0.15% dry weight, *P* < 0.001, Table 1).

**Table 1:** The nutritional composition (% dry weight) of the feces of sea urchins fed kelp (KF feces), the feces of sea urchins fed prepared feed (FF feces) and the sea cucumber feed (commercial feed mix with sea mud = 1: 6). Different letters indicates significant differences among the three diet (*P* < 0.05).

Diet	Nutritional composition		
	Crude fiber	Crude fat	Crude protein
KF feces	21.31 ± 0.98%^a^	0.62 ± 0.11%^a^	2.42 ± 0.37%^a^
FF feces	10.02 ± 0.32%^b^	0.80 ± 0.02%^ab^	14.31 ± 1.00%^b^
Sea cucumber feed	2.15 ± 0.15%^c^	1.40 ± 0.03%^b^	3.43 ± 0.17%^a^

## Discussion

In recent years, mass mortality of cultured sea cucumbers has occurred frequently in summer, causing on average about 30% of the lost income of *A. japonicus* aquaculture^4,5^. Therefore, improving the survival rate in summer is an important consideration for sea cucumber aquaculture^5^. In the present study, *A. japonicus* fed KF feces had significantly higher survival rate than those of the feed group, suggesting the feces of sea urchins fed kelp is a promising food for sea cucumber aquaculture in summer. Further, sea cucumbers in groups S and FF had no significant difference in survival rate. Therefore, although the feces of sea urchins fed prepared feed could be used as a suitable food source for improving the survival for *A. japonicus* at the optimum temperature and in winter^20^, the prepared feed feces has no advantage in improving survival in summer.

Although *A. japonicus* normally survive over a large temperature range of 30 °C^4,23,24^, rapid changes in water temperature could trigger stress on sea cucumbers and even cause death^25–28^. CT_max_ is the temperature at which an organism loses muscle coordination in response to external stimulation^6,29^. *Apostichopus japonicus* fed KF fecal diet showed significantly higher CT_max_ than those fed sea cucumber feed, suggesting the KF fecal diet enhances the resistance of sea cucumbers to high temperature. Besides, water temperature extends beyond a direct effect on sea cucumbers^9,30^. It leads to bacterial infectious diseases^31^. High temperature increases the activity of *Vibrio*, and thus causes the high incidence of skin ulceration syndrome (one of the most serious bacterial diseases of *A. japonicus*) in summer^32,33^. Our results showed that the sea cucumbers fed the KF feces had no skin ulceration, which was significantly lower than the incidence of skin ulceration of sea cucumbers in the other two groups when being exposed to the same infectious solution (made by using sea cucumbers that had died of the skin ulceration syndrome). Ingesting feces of sea urchins fed kelp, which is rich in bacteria and digestive enzymes, probably enhances the resistance, and immune ability of *A. japonicus*^34,37^. Further, the pathogens usually attack individuals with low immunity, and are hardly pathogenic to healthy individuals^5^. Better resistance to high temperature (an increase in CT_max_) and pathogen attack provided by the feces of sea urchin fed kelp further support the present result of the highest survival, suggesting that this kind of feces is an effective food to solve the summer problem and prevent mass mortality in summer.

Food consumption is another important factor that is greatly related to aquaculture efficiency of *A. japonicus* in summer^7^. Sea cucumbers of the FF group had the highest food consumption on fresh FF feces in all food consumption tests, indicating fresh FF feces of sea urchins is an appropriate food in summer. Fresh FF feces of sea urchins may be more nutritious and/or meet more nutritional requirements of sea cucumbers^22,35,36^. However, this kind of feces became less suitable for sea cucumbers after 24–48 h of ageing, since sea cucumbers of group FF had significant less fecal consumption when consumed feces aged 24 h at 25 °C, compared to the fresh feces of sea urchins fed prepared feed. This large reduction in fecal consumption implies that the feces of sea urchins fed prepared feed decays in a short time (within 48 h). This kind of feces appears to decay in 48–72 h of ageing time at 15 °C and over 72 h ageing at 5 °C^37^, but decays within 48 h at 25 °C. These results indicate that high temperature accelerated the deterioration of feces. Further, sea cucumbers of KF group had similar amount of food consumption on fresh KF feces and aged KF feces. The 24 h of ageing at 25 °C for the KF feces had no significant effects on fecal consumption, suggesting that this kind of feces does not decay in a short time (48 h). Different ageing processes and decay time are the results of the action of microorganisms and enzymes in different feces^11,12^. Considering that nutrient composition of feces would affect this process, we measured the nutritional concentration of the three foods, and found the highest concentration of protein (~ 15% dry weight), a low fat (~ 1% dry weight), high ash (~ 74% dry weight) and low fiber concentration (~ 10% dry weight) in the feces of sea urchins fed the prepared feed. The high protein concentration could explain why this feces deteriorated in 24–48 h, because proteins deteriorate more quickly than fiber at high temperatures^38^. The spoiled proteins would trigger harmful bacteria, and thus cause deterioration of water quality and massive mortalities^38^, which explains the low survival of sea cucumbers. The concentration of crude fiber is the highest (~ 22% dry weight) in feces of sea urchins fed kelp among the three diets. As feces ages, feces may become more nutritional, palatable and absorbable owing to pre-processing function of microorganisms on fibers^11^. The nutrient composition, which is high in fiber, low in fat (~ 0.6% dry weight) and protein concentration (~ 2.5% dry weight), may be responsible for the non-decay in a short time (48 h) for the KF feces. Food consumption on the fresh sea cucumber feed has no significant difference with that of feed aged for 24 h. This can be explained by the absence of active substances in the fresh dry sea cucumber feed.

We further evaluated the growth of sea cucumbers over a 5-week culture period for the great relation between feeding, the nutrition value of food, and growth^39^. Sea cucumbers fed fecal diets (~ 30% of group FF, ~ 28% of group KF) had a higher individual growth rate than those fed prepared feed (~ 15%), suggesting the fecal diets have a great potential in improving individual growth in summer. The protein-rich feces of sea urchins fed prepare feed can meet the nutrients needed to grow sea cucumbers, according to individual growth rate and earlier experimental results of the good growth of sea cucumbers in winter and at 15 °C^20^. Yet the productivity needs to take into account the overall efficiency, including survival and growth in summer. Due to the 100% survival, weight gain of sea cucumbers fed the KF feces of sea urchins is the highest among three groups. The feces of sea urchins fed kelp is more suitable for reducing mortality and addressing the problems and achieving higher efficiency in the *A. japonicus* aquaculture in summer.

## Methods

### Animals

*Apostichopus japonicus* were transported from Dalian Xinyulong Ecological Seedling Industry Co., Ltd. to the Key Laboratory of Mariculture & Stock Enhancement in the North China’ s Sea. They were temporarily cultured in a tank (length × width × height: 75 × 45 × 40 cm) with a temperature-controlled system (Huixin Co., Dalian, China). They were fed sea cucumbers commercial feed (An yuan Industry Co., Ltd.) with sea mud (1: 6) until the experiment started on May 10, 2021.

The sea urchin *Strongylocentrotus intermedius* (test diameter = 26.74 ± 5.41 mm) from the Key Laboratory of Mariculture & Stock Enhancement in the North China’ s Sea, Ministry of Agriculture and Rural Affairs, Dalian Ocean University were maintained in two fiberglass tanks (length × width × height: 75 × 45 × 40 cm; HXSWT-101, Huixin Co., Dalian, China) with temperature-controlled systems at 25 °C without feeding for five days. The sea urchins from each tank were randomly separated and cultured in 6 cylindric cages (20 cm in diameter, 0.5 cm mesh size) at 25 °C and fed the fresh brown alga *Saccharina japonica *ad libitum and prepared sea urchin feed ad libitum in two tanks, respectively. The plastic grate at the bottom of the cages kept the food and sea urchins in the cages, but allowed the feces to pass through. The feces of sea urchins fed kelp (KF feces), and feces of sea urchins fed feed (FF feces) from the two tanks were removed by siphoning and collected or cleaned daily at 9:00 am. There was no major variation in water temperature (25.0 ± 1.0 °C) and salinity (30.68 ± 0.36‰) of the three tanks during the acclimation period, according to the daily measurement with an YSI probe (YSI Incorporated, OH, USA). The photoperiod was 12D: 12L. The seawater was renewed daily.

### Experimental design

**Figure 5:**
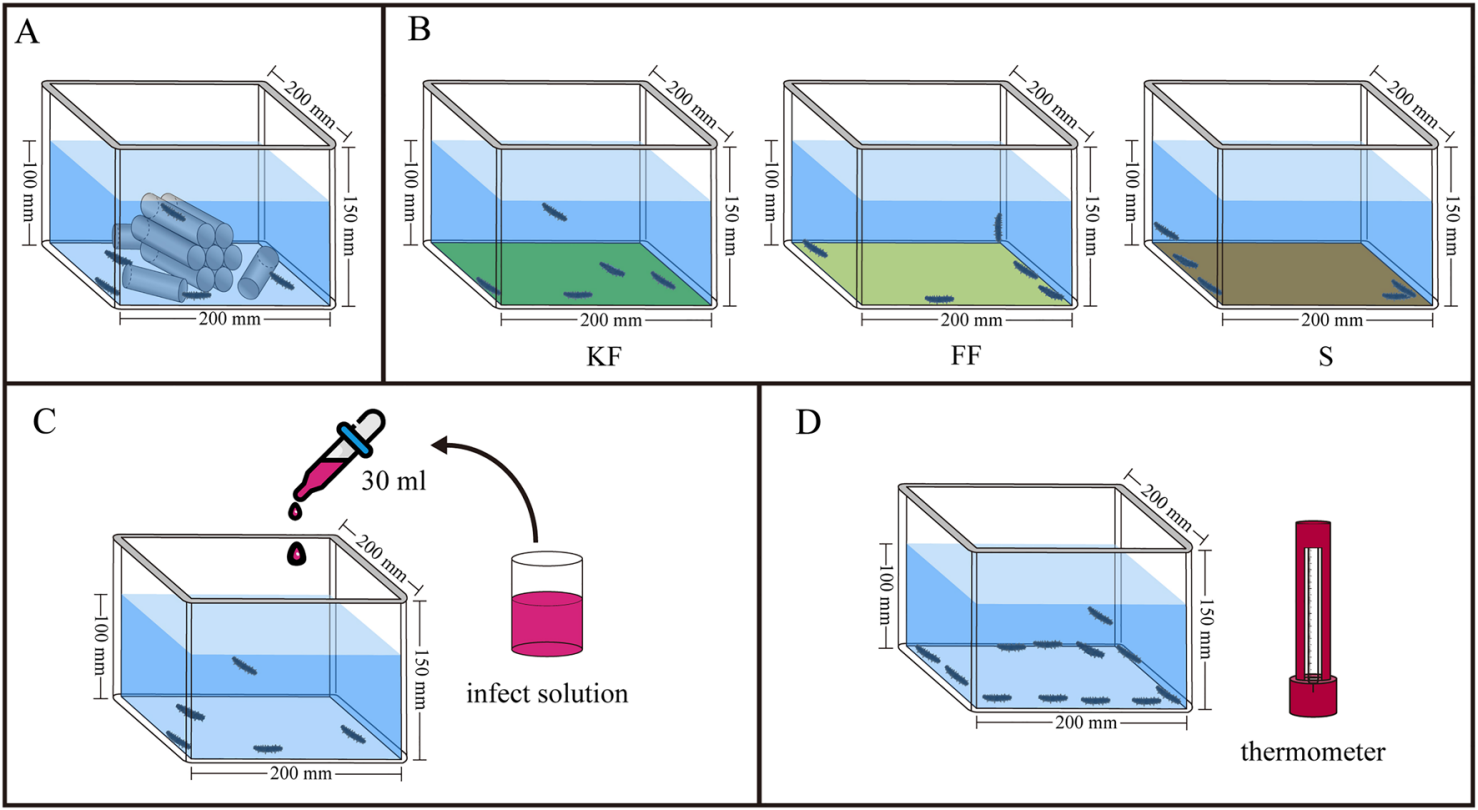
The conceptual diagrams showing the containers for culture (**A**), feeding consumption experiment (**B**), disease challenge assay (**C**), and thermal tolerance (**D**). KF, FF, S refer to KF feces (the feces of sea urchins consumed kelp), FF feces (the feces of sea urchins consumed feed) and the sea cucumber feed (commercial feed mix with sea mud = 1: 6), respectively.

Three diets were set as the experimental factor: KF feces (group KF), FF feces (group FF) and commercial feed of sea cucumbers (Anyuan Industry Co., Ltd.) with sea mud (1: 6) (group S) for the three group of *A. japonicus*. Each of 64 sea cucumbers (body weight = 2.79 ± 0.66 g) were randomly put into eight experimental plastic devices at 25 °C (length × width × height: 20 × 20 × 15 cm, with fixed shelters on the bottom) (Fig. 5A) for one group. The five weeks of culturing was conducted from 10 May 2021 to 14 June 2021.

Water temperature was 25 ± 0.5 °C, controlled by a temperature-controlled systems (Huixin Co., Dalian, China). The salinity was 30.9 ± 0.72‰ and dissolved oxygen was 6.7 ± 0.3 g/mL (mean ± SD), according to daily measurement with a YSI probe (YSI Incorporated, OH, USA). The oxygenated seawater was renewed every two days.

The number of dead *A. japonicus* was recorded after the five-week culturing period. Survival rate was calculated as the number of survived individuals divided by the number of all involved sea cucumbers. Then we measured the food consumption, growth performance, and subsequently preformed disease challenge and thermal tolerance tests, to investigate whether the fecal diets provide benefits to sea cucumbers.

### Food consumption

Food consumption was measured after the five-week experiment. Five sea cucumbers were placed in one plastic device (length × width × height: 20 × 20 × 15 cm, Fig. 5B). First, we fed them with corresponding food (5 g wet KF feces, 5 g wet FF feces, and 5 g sea cucumber feed) and cultured for 24 h at 25 °C using the method of water bath in three temperature-controlled tanks (length width height: 75 × 45 × 35 cm, HXSWT-101, Huixin Co., Dalian, China). The remained feces and remained sea cucumber feed were collected, dried and weighted after 24 h. We subsequently renewed the water and continued the experiment with 5 g wet KF feces aged for 24 h, 5 g wet FF feces aged for 24 h, and 5 g sea cucumber feed aged for 24 h in sea water. The remained feces and remained sea cucumber feed were collected, dried and weighted after 24 h (48 h after the experiment began). The whole experiment had eight replicates using different sea cucumbers for each group (N = 8). Sea cucumbers were put back for the following experiments after the harmless experiment of food consumption.

Food consumption was measured as follow^40^:

$${\text{Food}}\;{\text{consumption}} = {{\text{A}}_{1} - {\text{A}}_{0}}$$

Food consumption = average food consumption per 5 sea cucumbers (g/24 h), A_0_ = weight of provided food (g dried weight), A_1_ = weight of uneaten food (g dried weight). To avoid inactivate the active substances on feces before food composition, we respectively drying another three samples of KF feces and FF feces (wet weight = 5 g) as the A_0_ for the groups KF and FF.

### Growth

Sea cucumbers were drained in a net for one minute and then weighted using an electric balance (G & G Co., USA) for all the groups at the end of the experiment (N = 8). Individual growth rate was calculated according to the following formula^41^:

$$\text{Individual\,growth\,rate }=\left(\overline{{\text{W} }_{\text{t}}}-\overline{{\text{W} }_{0}}\right)\times 100{\%}$$

Weight gain rate was calculated according to the following formula^42^:

$${\text{WGR}} = \frac{{({\text{W}}_{{\text{t}}} {\text{ - W}}_{0} )}}{{{\text{W}}_{0} }} \times 100{\% }$$

W_t_ is terminal wet weight of sea cucumbers (g); W_0_ is initial wet weight of sea cucumbers (g).

### Disease challenge assay

Skin ulcerative syndrome is a highly infectious disease caused by bacterial infection in *A. japonicus*^43^. To observe the resistance of sea cucumbers fed different diets to the skin ulceration syndrome, we collected the carcasses of 20 sea cucumbers that died of the skin ulceration syndrome and mixed with 600 mL of seawater for 24 h as an infectious solution. Twenty healthy sea cucumbers were individually placed into 4 containers (length × width × height: 20 × 20 × 15 cm, each containing 4 L seawater; Fig. 5C) for each group (N = 20, Fig. 5C). Thirty mL of infectious solution was then injected into each box. We recorded the condition of sea cucumbers 48 h later using a digital camera (Legria HF20; mage Canon, Tokyo, Japan) and used IamgeJ (version 1.51n) to measure the area of ulcers or white spots on the skin of each sea cucumber. The area of ulcers per sea cucumber was calculated as follows:

$${\text{Skin}}\;{\text{ulceration}}\;{\text{proportion}}\;(\% ) = \frac{{{\text{S}}_{{\text{t}}} }}{{\text{S}}} \times 100{\%} $$

S_t_ = the area of ulcers or white spots of each sea cucumbers (cm^2^), S = the area of each sea cucumbers (cm^2^).

### Thermal tolerance

Twelve healthy sea cucumbers were randomly selected and placed in one plastic tank (length × width × height: 75 × 45 × 35 cm, HXSWT-101, Huixin Co., Dalian, China, N = 12, Fig. 5D) for each group. Water temperature increased from 25 °C to the lethal temperature by 1 °C hour^-1^. The temperature at which sea cucumbers showed any sign as ulcers, white spots, no response to external stimulation or evisceration was recorded as the CT_max_.

### Nutritional composition of diets

Samples of fresh KF feces and FF feces and sea cucumbers feed were collected to measure their organic composition (crude protein, crude fiber and crude fat) (N = 3, 50 g dry weight for each sample). Semi-micro Kjeldahl nitrogen method was used to measure the crude protein concentration of the two kinds of dried feces and feed. Digestion, distillation, absorption and titration were used during the included procedures^44^. The dried samples of each diet were boiled with a mixed solution (1.25% dilute acid and dilute alkali) for 30 min to measure their crude fiber concentration^45^. Soxhlet method was performed to assess the crude fat concentration of the three diets. About 10 g of samples were ashed at 550 °C for 48 h to measure the ash concentration of three diets^45^.

### Statistical analysis

Normal distribution and homogeneity of variance were assessed using the Kolmogorov–Smirnov test and Levene's test, respectively. Kruskal–Wallis test was used to compare the survival rate between the three groups. Mann–Whitney U test was preformed to compare the food consumption within the groups FF and KF, and the independent T-test was used to compare the food consumption within the group S and the fiber proportions among three diets. One-way ANOVA were used to compare the WGR, individual growth rate, CT_max_ and the crude protein among the three groups. Kruskal–Wallis test was performed to compare the differences of skin ulceration proportion and the crude fat among the three groups, because the data were non-normal and/or lacked homogeneity in the variance. All data analyses were performed using SPSS 19.0 statistical software. A probability level of *P* < 0.05 was considered being significant.

## Supplementary Information

Supplementary Information.

## Data Availability

All data generated or analyzed during this study are included in Supplementary Information file.
