# Supplementary material for: The feces of sea urchins as food improves survival, growth, and resistance of small sea cucumbers Apostichopus japonicus in summer
Source: Sci Rep. 2023 Apr 1;13:5361. doi: 10.1038/s41598-023-32226-y (PMC10067838; doi:10.1038/s41598-023-32226-y)
Supplement: Supplementary file 1 — Supplementary Information. [file 41598_2023_32226_MOESM1_ESM.docx]

**The feces of sea urchins as food improves survival, growth, and** **resistance of small sea cucumbers *Apostichopus japonicus* in summer**

Yushi Yu, Peng Ding, Yihai Qiao, Yansong Liu, Xiajing Wang, Tongdan Zhang, Jun Ding, Yaqing Chang, Chong Zhao^*^

Key Laboratory of Mariculture & Stock Enhancement in North China’s Sea, Ministry of Agriculture and Rural Affairs, Dalian Ocean University, Dalian, China

**Table S1.** Data of survival rate of the three group after 5-weeks culture (mean ± SD, N = 8). KF, FF, S refer to group KF (consume the feces of sea urchins fed kelp), group FF (consume the feces of sea urchins fed prepared feed) and group S (consume the sea cucumber feed).

| **Group** | **KF** | **FF** | **S** |
| --- | --- | --- | --- |
| Survival rate | 100 ± 0.00 % | 84.38 ± 8.84 % | 91.06 ± 9.11 % |

**Table S2.** Data of food consumption of the three group after 5-weeks culture (mean ± SD, N = 20). KF, FF, S refer to group KF (consume the feces of sea urchins fed kelp), group FF (consume the feces of sea urchins fed prepared feed) and group S (consume the sea cucumber feed).

| **Group** | **KF** | **FF** | **S** |
| --- | --- | --- | --- |
| Fresh food | 3.29 ± 0.42 g | 3.94 ± 0.20 | 2.75 ± 0.26 |
| Food aged for 24h | 3.57 ± 0.41 g | 2.53 ± 0.44 | 2.80 ± 0.25 |

**Table S3.** Data of weight gain rate (WGR) and individual growth rate of the three group after 5-weeks culture (mean ± SD, N = 12). KF, FF, S refer to group KF (consume the feces of sea urchins fed kelp), group FF (consume the feces of sea urchins fed prepared feed) and group S (consume the sea cucumber feed).

| **Group** | **KF** | **FF** | **S** |
| --- | --- | --- | --- |
| Weight gain rate | 27.43 ± 5.60% | 30.46 ± 5.50% | 15.09 ± 2.56% |
| Individual growth rate | 28.40 ± 6.75% | 0.02 ± 11.12% | 7.83 ± 6.55% |

**Table S4.** Data of CT_max_ of the three group after 5-weeks culture (mean ± SD, N = 12). KF, FF, S refer to group KF (consume the feces of sea urchins fed kelp), group FF (consume the feces of sea urchins fed prepared feed) and group S (consume the sea cucumber feed).

| **Group** | **KF** | **FF** | **S** |
| --- | --- | --- | --- |
| CT_max_ | 35.90 ± 0.38 °C | 35.33 ± 0.74 °C | 34.52 ± 0.87 °C |

**Table S5.** Data of skin ulceration proportion of the three group after 5-weeks culture (mean ± SD, N = 20). KF, FF, S refer to group KF (consume the feces of sea urchins fed kelp), group FF (consume the feces of sea urchins fed prepared feed) and group S (consume the sea cucumber feed).

| **Group** | **KF** | **FF** | **S** |
| --- | --- | --- | --- |
| **Skin** ulceration proportion | 0.00 ± 0.00 % | 27.15 ± 9.18 % | 24.90 ± 9.00 % |

**Table. S6** The results for normal distribution and homogeneity of variance of all data and consequent statistical analysis.

| Index | Normal distribution | Homogeneity | Methods of statistical analysis | Significance |
| --- | --- | --- | --- | --- |
| Survival rate | *P* < 0.05 | - | Kruskal-Wallis | *P* = 0.002 |
| Food consumption | group KF *P* < 0.05 | - | Mann-Whitney U | *P* = 0.161 |
|  | group FF *P* < 0.05 | - | Mann-Whitney U | *P* < 0.001 |
|  | group S *P* > 0.05 | - | Independent-samples T test | *P* = 0.707 |
| Individual growth rate | *P* > 0.05 | *P* = 0.194 | one-way ANOVA | *P* < 0.001 |
| WGR | *P* > 0.05 | *P* = 0.021 | one-way ANOVA | *P* < 0.001 |
| Skin ulceration proportion | *P* < 0.05 | - | Kruskal-Wallis | *P* = 0.008 |
| CTmax | *P* > 0.05 | *P* = 0.024 | one-way ANOVA | *P* < 0.001 |
| Crude protein | *P* > 0.05 | *P* = 0.089 | one-way ANOVA | *P* < 0.001 |
| Crude fat | *P* < 0.05 | - | Kruskal-Wallis | *P* = 0.031 |
| Crude fiber | *P* > 0.05 | *P* = 0.162 | one-way ANOVA | *P* < 0.001 |
